# Root and Canal Morphology of Mandibular Premolars in a Saudi Subpopulation: A Cone-Beam Computed Tomography Study

**DOI:** 10.1155/2022/4038909

**Published:** 2022-03-08

**Authors:** Saad M. Al-Zubaidi, Moazzy I. Almansour, Ahad S. Alshammari, Nada N. Al Mansour, Ahad F. Alshammari, Yazeed S. Altamimi, Ahmed A. Madfa

**Affiliations:** ^1^Department of Restorative Dental Science, University of Ha'il, Ha'il, Saudi Arabia; ^2^Ministry of Health, Ha'il, Saudi Arabia

## Abstract

**Objectives:**

The efficacy of root canal therapy is dependent on a thorough understanding of both normal and aberrant root canal morphology. As a result, the purpose of this study was to use CBCT to characterize the exact root and canal morphology of mandibular premolars in a Saudi subpopulation.

**Methods:**

The current study included 1000 mandibular premolars (507 first premolars and 493 second premolars) with completely developed roots. CBCT was performed to assess the shape of the roots and to classify the canal anatomy according to Vertucci's classification. The incidence and similarity of the left and right sides, as well as men and women, were investigated. The data were examined using the chi-square test.

**Results:**

Of the 507 mandibular first premolars analyzed, 484 (95.5%) had one root, whereas 23 (4.5%) had two roots. Of the 493 mandibular second premolars analyzed, 489 (99.2%) had one root, whereas four teeth had two roots (0.8%). There were no statistically significant variations in the number of roots identified across groups (*p* > 0.05). The most prevalent in mandibular first premolars was type I, accounting for 70.0% (*n* = 355) of the studied sample, followed by type II (14.2%, *n* = 72) and type IV (10.1%, *n* = 51). For mandibular second premolar, type I had the highest incidence (449 (91.1%)), followed by type II (5.7%, *n* = 28).

**Conclusion:**

In a Saudi subpopulation, the majority of mandibular first and second premolar teeth had a single root with a type I canal system. On the other hand, numerous roots with various canal classifications were found.

## 1. Background

The root canal shape varies from tooth to tooth. Many studies revealed that the root canal is a complicated system that divides and connects canals along the way to the apex, rather than a single canal that runs continuously from opening to apex [1–3].

The aim of endodontic therapy is to achieve thorough shaping and cleaning of all pulp spaces and their complete obturation with an inert filling material. A thorough grasp of pulp anatomy is required for root canal treatment effectiveness, and a lack of this information might result in treatment failure [4, 5]. Endodontic performance necessitates a detailed knowledge of the pulp's natural and atypical arrangements, as well as possible alterations. Forming a mental image of the dental pulp from the coronal part to the apical foramen is critical for an operator. Every canal has irregular and secret areas that must be considered during endodontic care. To prevent or mitigate treatment failure, chemomechanical cleaning and shaping must be able to enter these secret regions and clean and form them as thoroughly as possible [4–9].

Mandibular first and second premolars have similar morphology, and these single-rooted premolars have single root canal types. On the other hand, reports show that root canal morphology in premolars is more difficult than it appears on simple radiographs. Extra canals and a number of canal configurations can be found in several roots [10, 11]. Mandibular first and second premolars are among the most problematic teeth to treat in the mouth. The most possible explanation is that clinicians failed to notice the various differences in canal morphology that these teeth may have. A disproportionate number of cases may result in flare-ups and/or failures if the entire root canal system is not found and properly handled [10, 11]. For the identification and proper treatment of teeth with various anatomical differences, a detailed understanding of root canal anatomy, careful radiographic analysis, and proper adjustment of the traditional access cavity preparation tend to be necessary. Before beginning root canal care, it is important to consider the possibility of root canal morphology variations [12].

Cone-beam computed tomography (CBCT) is a noninvasive tool that can help clinicians analyze maxillofacial anatomy in sagittal, axial, and coronal sections while also providing high-quality 3D diagnostic images without overlapping structures [13]. The number of canals, as well as their separation or convergence from one another, can all be imagined in 3D. For these purposes, CBCT is recommended for accurate root canal system evaluation [14].

The effectiveness of root canal care is based on a detailed understanding of both normal and abnormal root canal anatomy. Working knowledge of such information is needed for proper shaping, cleaning, and obturation of the root canal system in three dimensions [14, 15]. The occurrence of missing roots or canals in teeth that required retreatment was 42%, according to Hoen and Pink [16]. The number of roots and root canals of mandibular premolars varied, and the internal root canal shape might be extremely diverse [17]. The broad variety in root canal anatomy of these teeth makes conducting effective endodontic procedures the most complicated of all [18]. Several studies [19–22] reported that a high ratio of mandibular first premolars had multiple canals. Different ethnic or geographic groups can have different rates, locations, and morphologies of root canal systems [19–22]. Mandibular premolars with two or more canals were observed in 13.7% of American people [19] and 46% of the Chinese population [20].

The current study's goal was to employ CBCT to determine the accurate root anatomy, as well as root canal configuration, of mandibular first and second premolars in a Saudi subpopulation.

## 2. Method

The Medical Ethics Committee of the College of Dentistry at the University of Hail in Saudi Arabia accepted the study's protocol. Due to the retroactive character of the investigation, the Ethics Committee of the College of Dentistry at the University of Hail waived informed consent. The patients who had CBCT scanning to diagnose mandibular premolars were examined. The collected data were subjected to a retrospective examination. All of the data related to patients who were seen at the Hail clinics between May 2017 and November 2020 were updated.

A database including 3000 CBCT images was examined. Accurate CBCT images of mandibular premolars that had totally grown roots in individuals aged 18–60 years satisfied the inclusion criteria and were included in the current investigation. Teeth with periapical disorders and low-quality CBCT scans were excluded. Additionally, teeth having calcification or resorption of roots were also omitted. Furthermore, endodontically treated teeth, postcoronal restorations, metallic restorations, crown restorations, and scan artifacts were all left out [23]. Anatomical symmetry was evaluated by comparing images from both sides with teeth. After analyzing 3000 scans for inclusion/exclusion criteria, the final specimen size for this investigation was 1000 mandibular premolars.

The Carestream CS 8100 3D CBCT equipment was utilized for the scans (Carestream Dent LLC, Atlanta, USA). The X-ray generator has a voltage range of 60–90 kV, a current range of 2–15 mA, and a frequency range of 140 kHz. This machine's specifications included a CMOS sensor with Dental Volumetric Reconstruction (DVR), a scan period of 3 to 15 seconds, fields of view (FOV) of 4 × 4, 5 × 5, 8 × 5, and 8 × 8 cm, and a voxel size of 75 m minimum. The pictures were analyzed using the CS 3D Imaging Software (Carestream Dent LLC, Atlanta, USA).

Four examiners received calibration training prior to the evaluation. To verify the accuracy of the findings of this study, we randomly selected 40 CBCT images to evaluate interexaminer reliability by detecting root canal counts and identifying the kind of root canal type based on Vertucci classification. The reliability of intraexaminer and interexaminer was examined.

The results were evaluated using version 22.0 of the Statistical Package for Social Sciences (SPSS Inc., Chicago, IL, USA). The total number of roots, root canal configuration, unilateral and bilateral events, and root canal configuration were all studied. The incidence and similarity of the left and right sides, as well as women and men, were investigated. The findings were evaluated by using the chi-square test. At the threshold of *p* = 0.05, statistical significance was determined.

## 3. Results

The total number of mandibular first premolars that were included in the present study was 507 (50.7%). However, for mandibular second premolars, the number of scanned teeth was 493 (49.3%).

Table 1 shows the number of roots on the right and left sides in relation to gender and tooth location. Of the 1000 teeth assessed, 973 mandibular premolars had one root (97.3%), whereas 27 (2.7%) were two-rooted. There was no statistically significant difference (*p* > 0.05) between gender and the number of roots or tooth position and the number of roots.

Table 2 shows the number of roots in relation to tooth type. Of the 500 mandibular first premolars examined, 484 teeth were single-rooted (95.5%), whereas 23 (4.5%) had two roots. For mandibular second premolars, 489 premolars had one root (99.2%), while 4 teeth had two roots (0.8%). There were substantial variations in the number of roots discovered across groups (*p* > 0.05).

**Figure 1:**
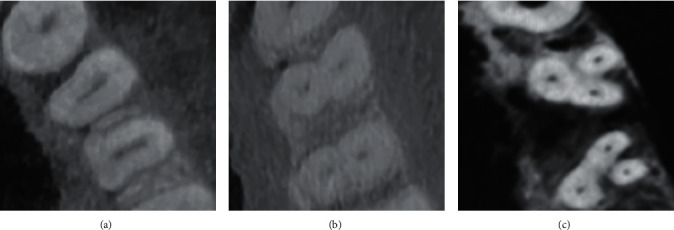
Different variations of root canal types in mandibular first premolar. (a) One canal; (b) two canals; (c) three canals.

**Figure 2:**
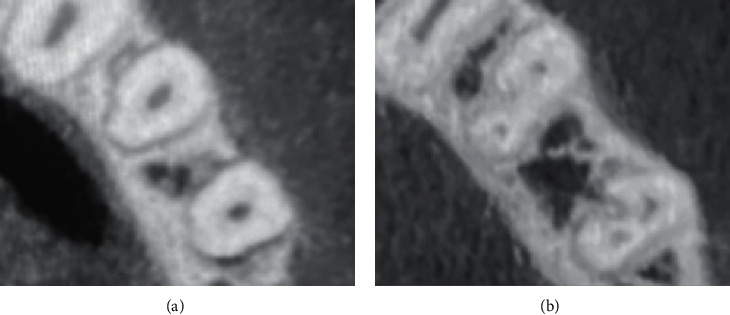
Different variations of root canal types in mandibular second premolar. (a) One canal; (b) two canals.

Figures 1 and 2 show different variations of root canal types in mandibular premolars. This study discovered differences in root canal types according to Vertucci's categorization. Type I was the most common for mandibular first premolars, accounting for 70.0% of the studied sample (*n* = 355), followed by type II (14.2%, *n* = 72) and type V (10.1%, *n*=(51). 11 of specimens (2.2%) had Vertucci type III, while either type VII or type VIII were only detected in 2 samples (0.4%) as revealed in Table 3. Type I had the highest occurrence (449 (91.1%)) of mandibular second premolar, followed by type II (5.7%, *n* = 28). Fourteen specimens (2.8%) were type IV, whereas either type III or type VI were only observed in one sample (0.2%) as displayed in Table 3.

## 4. Discussion

The root canal structure is a portion of the tooth that the dentist cannot directly see. Radiographs are useful for visualizing root canal structure, but they are restricted in that they only display two-dimensional representations of a three-dimensional object [23]. As a result, correct root anatomy information is critical to radiographic tools, tactile sense, and experience of the operator, all of which contribute to endodontic treatment success. Endodontic therapy failure is often caused by incomplete disinfection of the root canal system [24]. Adequate disinfection is very important because neither technique could completely obturate the root canal system [25].

In clinical instances, conventional straight-on or even oblique radiography may obscure the complex root canal anatomy of mandibular premolars. An earlier investigation [26] found that buccolingual or mesiodistal angulated radiographs had poor sensitivity in detecting root canal anatomy. Traditional periapical radiographs are a valuable clinical diagnostic method for determining root canal anatomy. They do, however, contain defects such as deformation and superimposition of dental features [27]. In these cases, CBCT has been proposed as a way to get a 3D confirmatory diagnosis without damaging the teeth. It has high resolution and is well suited for use as an alternative to traditional radiography in endodontic applications [28]. Traditional periapical radiography combined with CBCT may be applied to assess or approve the existence and position of canal bifurcation while diagnosing canal discrepancies or detecting a shift in direction/shape in the middle-apical third of the canal.

The current report has revealed enormous variations in the anatomy of root and root canal of mandibular first and second premolars among a Saudi subpopulation. Since the root canal configurations have an influence on the results of root canal care, physicians must be aware of the anatomical complications that exist. Missed canals, perforations, and canal transportations are all examples of iatrogenic operational errors caused by a lack of understanding of root canal morphology.

Mandibular first premolars are among the most difficult teeth to endodontically treat due to their varied root canal forms and restricted access to a second canal [1]. According to the current analysis, single-rooted premolars account for 95.5% of mandibular first premolars, followed by double-rooted (4.5%) and finally three-rooted premolars (0.0%). The results of this study are consistent with those of Alfawaz et al. [29], who discovered one root (96.4%), two-rooted (3.1%), and three-rooted (0.5%). As compared to other research in Turkey (0%) [30], Iran (2%) [31], and Jordan (3%) [32], the occurrence of mandibular first premolars with two roots was more in this sample. Root canal morphology investigations of removed teeth are considered intrusive research when compared to CBCT. When the clearing technique was used, a greater frequency was identified in Caucasians (10.9%) via radiographs [19], Egyptians (3.2%) [33], South Asians (6%) [34], and South Saudi subpopulations (18%) [35]. Furthermore, an analysis of a Kuwaiti population using conventional radiographic examination found a high occurrence of mandibular first premolar teeth with two roots (15%) [36].

Type I canal system was found in the highest number of mandibular first premolars, followed by type II and type IV. In two teeth, Type VIII canal morphologies with three distinct canals were noticed. According to Vertucci's categorization [37], type I canal configuration was more prevalent (67.39 percent) than the other canal configurations. Multiple canals have been identified in mandibular first premolars from 0.2% to 39.5% [24, 33, 35, 38, 39]. Velmurugan and Sandhya [40] found that type II canal configuration was in 16.6% of cases, while Parekh et al. [41] found that it was in 5% of cases. The prevalence of type II was stated to be 3.6% by Alfawaz et al. [29]. Another examination [35] found that type I was found in 69% of the Saudi population, which was consistent with the current study (70%).

Several research studies have exposed that mandibular second premolars have two or more canals [2, 42, 43]. In the middle or apical third of the main root canal, the second canal is typically tiny and branches toward the lingual component [44]. This study found that 0.5% of mandibular second premolars had two canals, which is similar to the findings of Llena et al. [43] and Shetty et al. [45]. Conversely for the present study, Sert and Bayirli [42] observed a greater incidence of two canals in mandibular second premolars, accounting for 29% of in their research. This investigation included type I and IV configurations. Llena et al. [43] and Shetty et al. [2] have discovered a larger variety of variants with type I, II, III, V, and VIII configurations. Differences in study designs or ethnic variations may have an impact on the findings.

The most common root morphology in mandibular second premolars was one root, followed by double-rooted. In addition, the majority of these samples had type I canal, followed by type II and lastly type IV configurations. Our results were consistent with earlier reports, which revealed that the prevalence of type I canal configuration in a Turkish population was 93.63% and 98.5%, respectively [30, 46]. Cleghorn et al. [47] examined the root canal morphology in mandibular second premolars and discovered that 91% had one canal and 9% had two canals. Furthermore, the current study agrees with a previous study of a Saudi population, which found that type I incidence was 90.1%, followed by type II at 4.4%.

One root was noticed predominantly in mandibular premolars, 90.76% and 98.16%, correspondingly, and with one canal, 77.9% and 96%, correspondingly in a selected German population, which correlates with the findings of the present study [48]. Case reports for two-rooted mandibular second premolars with three root canals [49] and three roots with three root canals [50] have also been published.

According to Ok and their co-workers [45] and Bürklein and associates [48], men had slightly more roots and root canals in mandibular first premolars than women. Furthermore, Martins et al. [51] discovered that women had fewer roots and more type I canal configurations, whereas men had three root canal system types, with no gender difference. In the current study, there was no correlation (*p* > 0.05) between gender, degree of symmetry, and the number of roots in the first and second premolars. Both mandibular first and second premolars had a high degree of symmetry in our research.

The canal systems of mandibular first premolars in a Saudi subpopulation showed a lot of variation and complexity. Mandibular second premolars had less variation in root and canal configuration than mandibular first premolars. A CBCT scanner was able to detect these complex variations, which is important. This indicates that CBCT may be used as an additional method to increase the efficiency of root canal therapy in mandibular premolars with complex canal morphology. The increased failure rate that occurs when additional canals are missed during root canal therapy illustrates the importance of accurately assessing the presence of complex canal systems. When traditional radiography is inconclusive, CBCT scanning is extremely useful in detecting anomalous canal morphology.

This study has a few limitations that must be addressed. To fully represent the Saudi population, a larger sample size is advocated for future study. The age of the patients, as well as changes in FOV, should be taken into account in future research. Furthermore, the spatial resolution of the CBCT employed in this study was lower than that of micro- and nano-CT, which might have influenced the results. Further multicenter research employing improved methods such as micro-CT may be able to address the limitations of the current study.

## 5. Conclusions

Within the limitations of this study, it can be concluded that the patient's race is an irrefutable factor that determines the morphology of the root canal system. The root canal anatomy of mandibular premolars revealed significant differences among Saudi subpopulations. These findings suggest that practitioners should be aware of and avoid failures caused by additional missed canals. To achieve acceptable patient outcomes, practitioners must be aware of the intricacies of root canal architecture and employ the most up-to-date and precise armamentarium.

## Figures and Tables

**Table 1:** Number of roots for gender and tooth position in the studied sample.

Number of roots	Gender			Tooth position		
	Male	Female	Total	Left side	Right side	Total
One root *n* (%)	504 (50.4)	469 (46.9)	973 (97.3)	482 (48.2)	491 (49.1)	973 (97.3)
Two roots *n* (%)	16 (1.6)	11 (1.1)	27 (2.7)	15 (1.5)	12 (1.2)	27 (2.7)
Three roots *n* (%)	0 (0)	0 (0)	0 (0)	0 (0)	0 (0)	0 (0)

Chi-square, Fisher's exact tests; for gender *p* > 0.05; for side *p* > 0.05.

**Table 2:** Number of roots for gender and tooth position in mandibular first and second premolars.

Number of roots		Gender			Tooth position		
		Male	Female	Total	Left side	Right side	Total
First premolars	One root *n* (%)	250 (49.3)	234 (46.2)	484 (95.5)	238 (46.9)	246 (48.5)	484 (95.5)
	Two roots *n* (%)	14 (2.8)	9 (1.8)	23 (4.5)	13 (2.6)	10 (2.0)	23 (4.5)
	Three roots *n* (%)	0 (0)	0 (0)	0 (0)	0 (0)	0 (0)	0 (0)

Second premolars	One root *n* (%)	254 (51.5)	235 (47.7)	489 (99.2)	244 (49.5)	245 (49.7)	489 (99.2)
	Two roots *n* (%)	2 (0.4)	2 (0.4)	4 (0.8)	2 (0.4)	2 (0.4)	4 (0.8)
	Three roots *n* (%)	0 (0)	0 (0)	0 (0)	0 (0)	0 (0)	0 (0)

Chi-square, Fisher's exact tests; for gender *p* > 0.05; for side *p* > 0.05.

**Table 3:** Distribution of root canal types according to Vertucci's classification.

Type	I	II	III	IV	V	VI	VII	VIII
First premolar *n* (%)	355 (70.0)	72 (14.2)	11 (2.2)	51 (10.1)	14 (2.8)	0 (0)	2 (0.4)	2 (0.4)
Second premolar *n* (%)	449 (91.10)	28 (5.7)	1 (0.2)	14 (2.8)	0 (0)	1 (0.2)	0 (0)	0 (0)

## Data Availability

The data that support the results of this study are accessible from the corresponding author upon reasonable request.

## Abbreviations

FOV: Fields of view
DVR: Dental volumetric reconstruction
CBCT: Cone-beam computed tomography
3D: Three dimensional.

## References

[1] Gu Y., Lu Q., Wang H., Ding Y., Wang P., Ni L. (2010). Root canal morphology of permanent three-rooted mandibular first molars-Part I: pulp floor and root canal system. *Journal of Endodontics*.

[2] Filpo–Perez C., Bramante C. M., Villas-Boas M. H., Duarte M. A. H., Versiani M. A., Ordinola-Zapata R. (2015). Micro–computed tomographic analysis of the root canal morphology of the distal root of mandibular first molar. *Journal of Endodontics*.

[3] Tahmasbi M., Jalali P., Nair M. K., Barghan S., Nair U. P., Nair U. P. (2017). Prevalence of middle mesial canals and isthmi in the mesial root of mandibular molars: an in vivo cone-beam computed tomographic study. *Journal of Endodontics*.

[4] Corbella S., Baruffaldi M., Perondi I., Taschieri S. (2019). Surgically-oriented anatomical study of mandibular premolars: a CBCT study. *Journal of Clinical and Experimental Dentistry*.

[5] Corbella S., Baruffaldi M., Perondi I., Taschieri S. (2019). Cone-beam computed tomography investigation of the anatomy of permanent mandibular premolars in a cohort of Caucasians. *Journal of Investigative and Clinical Dentistry*.

[6] Walton R. E., Torabinejad M. (2002). *Principal and Practice of Endodontics*.

[7] Dennis D., Farahanny W., Prasetia W., Batubara F. Y. (2021). Biological and mechanical principles of chemomechanical preparation in root canal therapy: a review. *International Journal of Clinical Dentistry*.

[8] Siqueira J. F., Alves F. R. F., Almeida B. M., Machado de Oliveira J. C., Rôças I. N. (2010). Ability of chemomechanical preparation with either rotary instruments or self-adjusting file to disinfect oval-shaped root canals. *Journal of Endodontics*.

[9] McGurkin-Smith R., Trope M., Caplan D., Sigurdsson A. (2005). Reduction of intracanal bacteria using GT rotary instrumentation, 5.25% NaOCl, EDTA, and Ca(OH)_2_. *Journal of Endodontics*.

[10] Sundqvist G., Figdor D., Persson S., Sjögren U. (1998). Microbiologic analysis of teeth with failed endodontic treatment and the outcome of conservative re-treatment. *Oral Surgery, Oral Medicine, Oral Pathology, Oral Radiology & Endodontics*.

[11] Soares L. R., Arruda M., Arruda M. P. d. (2009). Diagnosis and root canal treatment in a mandibular premolar with three canals. *Brazilian Dental Journal*.

[12] England M. C., Hartwell G. R., Lance J. R. (1991). Detection and treatment of multiple canals in mandibular premolars. *Journal of Endodontics*.

[13] Patel S., Kanagasingam S., Mannocci F. (2010). Cone beam computed tomography (CBCT) in endodontics. *Dental Update*.

[14] Kaya-Büyükbayram I., Sübay R. l., Çolakoğlu G., Elçin M. A., Ordulu S. M. (2019). Investigation using cone beam computed tomography analysis, of radicular grooves and canal configurations of mandibular premolars in a Turkish subpopulation. *Archives of Oral Biology*.

[15] Sachdeva G. S., Ballal S., Gopikrishna V., Kandaswamy D. (2008). Endodontic management of a mandibular second premolar with four roots and four root canals with the aid of spiral computed tomography: a case report. *Journal of Endodontics*.

[16] Hoen M., Pink F. (2002). Contemporary endodontic retreatments: an analysis based on clinical treatment findings. *Journal of Endodontics*.

[17] Pedemonte E., Cabrera C., Torres A. (2018). Root and canal morphology of mandibular premolars using cone-beam computed tomography in a Chilean and Belgian subpopulation: a cross-sectional study. *Oral Radiology*.

[18] Slowey R. R. (1979). Root canal anatomy. Road map to successful endodontics. *Dental Clinics of North America*.

[19] Trope M., Elfenbein L., Tronstad L. (1986). Mandibular premolars with more than one root canal in different race groups. *Journal of Endodontics*.

[20] Lu T.-Y., Yang S.-F., Pai S.-F. (2006). Complicated root canal morphology of mandibular first premolar in a Chinese population using the cross section method. *Journal of Endodontics*.

[21] Zillich R., Dowson J. (1973). Root canal morphology of mandibular first and second premolars. *Oral Surgery, Oral Medicine, Oral Pathology*.

[22] Al-Zubaidi S. M., Almansour M. I., Al Mansour N. N. (2021). Assessment of root morphology and canal configuration of maxillary premolars in a Saudi subpopulation: a cone-beam computed tomographic study. *BMC Oral Health*.

[23] Yoshioka T., Villegas J., Kobayashi C., Suda H. (2004). Radiographic evaluation of root canal multiplicity in mandibular first premolars. *Journal of Endodontics*.

[24] Tabassum S., Khan F. R. (2016). Failure of endodontic treatment: the usual suspects. *European Journal of Dermatology*.

[25] Attavar S. H., Hegde M. N. (2021). Effect of irrigants and irrigating devices on disinfection of root canal system: a systematic review. *Journal of Advanced Oral Research*.

[26] Khedmat S., Assadian H., Saravani A. A. (2010). Root canal morphology of the mandibular first premolars in an Iranian population using cross-sections and radiography. *Journal of Endodontics*.

[27] Tzanetakis G., Lagoudakos T., Kontakiotis E. (2007). Endodontic treatment of a mandibular second premolar with four canals using operating microscope. *Journal of Endodontics*.

[28] Matherne R. P., Angelopoulos C., Kulild J. C., Tira D. (2008). Use of cone-beam computed tomography to identify root canal systems in vitro. *Journal of Endodontics*.

[29] Alfawaz H., Alqedairi A., Al-Dahman Y. H. (2019). Evaluation of root canal morphology of mandibular premolars in a Saudi population using cone beam computed tomography: a retrospective study. *The Saudi Dental Journal*.

[30] Calişkan M. K., Pehlivan Y., Sepetçioğlu F., Türkün M., Tuncer S. S. (1995). Root canal morphology of human permanent teeth in a Turkish population. *Journal of Endodontics*.

[31] Rahimi S., Shahi S., Yavari H. R., Manafi H., Eskandarzadeh N. (2007). Root canal configuration of mandibular first and second premolars in an Iranian population. *Journal of Dental Research, Dental Clinics, Dental Prospects*.

[32] Awawdeh L. A., Al-Qudah A. A. (2008). Root form and canal morphology of mandibular premolars in a Jordanian population. *International Endodontic Journal*.

[33] Alhadainy H. A. (2013). Canal configuration of mandibular first premolars in an Egyptian population. *Journal of Advanced Research*.

[34] Singh S., Pawar M. (2014). Root canal morphology of South Asian Indian mandibular premolar teeth. *Journal of Endodontics*.

[35] Chourasia H., Boreak N., Tarrosh M., Mashyakhy M. (2017). Root canal morphology of mandibular first premolars in Saudi Arabian southern region subpopulation. *Saudi Endodontic Journal*.

[36] Zaatar E. I., Al-Kandari A. M., Alhomaidah S., Al Yasin I. M. (1997). Frequency of endodontic treatment in Kuwait: radiographic evaluation of 846 endodontically treated teeth. *Journal of Endodontics*.

[37] Vertucci F. J. (1984). Root canal anatomy of the human permanent teeth. *Oral Surgery, Oral Medicine, Oral Pathology*.

[38] Green D. (1973). Double canals in single roots. *Oral Surgery, Oral Medicine, Oral Pathology*.

[39] Park J. B., Kim N., Park S., Kim Y., Ko Y. (2013). Evaluation of root anatomy of permanent mandibular premolars and molars in a Korean population with cone-beam computed tomography. *European Journal of Dermatology*.

[40] Velmurugan N., Sandhya R. (2009). Root canal morphology of mandibular first premolars in an Indian population: a laboratory study. *International Endodontic Journal*.

[41] Parekh V., Shah N., Joshi H. (2011). Root canal morphology and variations of mandibular premolars by clearing technique: an in vitro study. *The Journal of Contemporary Dental Practice*.

[42] Sert S., Bayirli G. (2004). Evaluation of the root canal configurations of the mandibular and maxillary permanent teeth by gender in the Turkish population. *Journal of Endodontics*.

[43] Llena C., Fernandez J., Ortolani P. S., Forner L. (2014). Cone-beam computed tomography analysis of root and canal morphology of mandibular premolars in a Spanish population. *Imaging Science in Dentistry*.

[44] Cleghorn B. M., Goodacre C. J., Christie W. H., Ingle J. I., Bakland L. K., Baumgartner J. C. (2008). Morphology of teeth and their root canal systems. *Ingle’s Endodontics*.

[45] Shetty A., Hegde M. N., Tahiliani D., Shetty H., Bhat G. T., Shetty S. (2014). A three-dimensional study of variations in root canal morphology using cone-beam computed tomography of mandibular premolars in a South Indian population. *Journal of Clinical and Diagnostic Research*.

[46] Ok E., Altunsoy M., Nur B. G., Aglarci O. S., Çolak M., Güngör E. (2014). A cone-beam computed tomography study of root canal morphology of maxillary and mandibular premolars in a Turkish population. *Acta Odontologica Scandinavica*.

[47] Cleghorn B., Christie W., Dong C. (2007). The root and root canal morphology of the human mandibular second premolar: a literature review. *Journal of Endodontics*.

[48] Bürklein S., Heck R., Schäfer E. (2017). Evaluation of the root canal anatomy of maxillary and mandibular premolars in a selected German population using cone-beam computed tomographic data. *Journal of Endodontics*.

[49] Al-Attas H., Al-Nazhan S. (2003). Mandibular second premolar with three root canals: report of a case. *Saudi Dental Journal*.

[50] Alenezi M., Tarish M., Alenezi D. (2015). Root canal treatment of three-rooted mandibular second premolar using cone-beam computed tomography. *Saudi Endodontic Journal*.

[51] Martins J. N. R., Marques D., Francisco H., Caramês J. (2018). Gender influence on the number of roots and root canal system configuration in human permanent teeth of a Portuguese subpopulation. *Quintessence International*.

